# Oculogyric Crisis in the Setting of Low Dose Risperidone and Benztropine Mesylate Use in a Patient With Schizophrenia: A Case Report and Review of Literature

**DOI:** 10.7759/cureus.27217

**Published:** 2022-07-25

**Authors:** Ariel Ruiz de Villa, Asad A Haider, Leora Frimer, Yvette Bazikian

**Affiliations:** 1 Internal Medicine, North Florida Regional Medical Center, Gainesville, USA

**Keywords:** ocular dystonia, fixed eyes, benztropine, risperidone, oculogyric crisis

## Abstract

In this case report, we describe a rather unique case of a 37-year-old male patient suffering from schizophrenia who presented with a fixed upwards gaze diagnosed as oculogyric crisis (OGC). This presentation was attributed to the effects of risperidone, a second-generation antipsychotic, while concomitantly taking benztropine mesylate. The latter is a medication commonly used to prevent dystonia in this type of patient population. Interestingly, the dose of risperidone was minimal, and side effects were not expected, making this presentation rare and not often cited or represented in the medical literature, given that second-generation antipsychotics are known to have a safer side effect profile when compared to their counterparts. We also aim to provide a review of the literature on this topic and describe the approach to diagnosis and treatment of such.

## Introduction

Oculogyric crisis (OGC) is the result of an acute dystonic reaction that isolates the eyes, resulting in an unpleasant and uncomfortable fixed gaze of the affected [1]. It is an uncommon but established adverse effect of first-generation antipsychotics [1]. The pathophysiology of OGC is thought to be due to the disruption of dopaminergic neurotransmission in the nigrostriatal pathway affecting the extraocular muscles [2]. This case is rather unique because second-generation antipsychotics, such as risperidone, are not often linked with OGC, especially when used in low doses [3,4]. Here we discuss the case of a 37-year-old male with schizophrenia being treated with low-dose risperidone and benztropine mesylate who presented from home with a fixed upwards gaze for several hours. Our patient was successfully treated and admitted to the hospital for further evaluation and surveillance. We aim to describe the presentation, approach to diagnosis, pathophysiology, and treatment of OGC while providing a review of the medical literature on this topic.

## Case presentation

The patient is a 37-year-old African American male with a past medical history of schizophrenia, polysubstance abuse, and several involuntary admissions to the inpatient behavioral health unit due to psychosis and intoxication. In the past, the patient received multiple treatments and counseling sessions for polysubstance use and intoxication with benzodiazepines, methylenedioxy-methylamphetamine (MDMA), cocaine, alcohol, and methamphetamines. 

During this encounter, the patient was brought from home via emergency medical services, activated by the patient’s mother, due to altered mental status. She was concerned about the patient being incoherent, agitated, withdrawn, and “looking upward all day”. She further reported that he did not respond despite physical and verbal stimuli, including vigorous shaking and a cold water shower.

**Figure 1:**
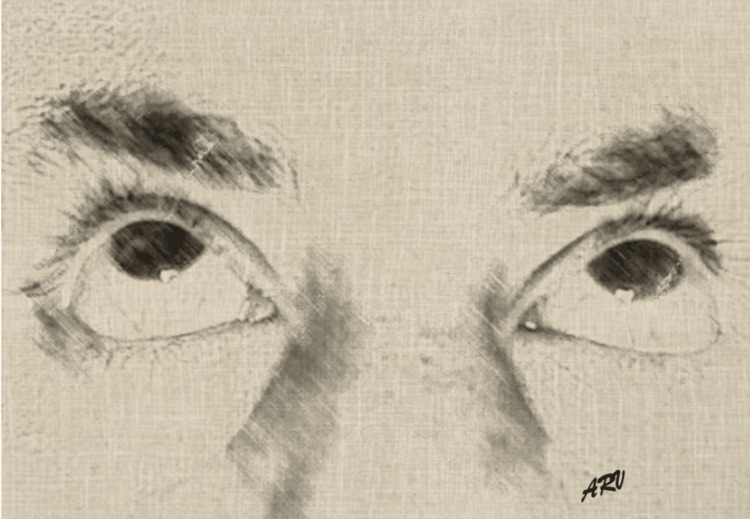
Sketch of oculogyric crisis Sketch portraying our patient’s oculogyric crisis, for illustrative purposes, not actual patient Artist rendering of oculogyric crisis: Ariel Ruiz de Villa, 2022

In the emergency department, the patient did not respond to questions and was not cooperative during the physical examination, intermittently pulling blankets over his head and making erratic upper and lower extremity movements. His neck was supple, and the patient had a fixed bilateral upwards gaze with an appropriate pupillary response to light. Figure 1 serves as a representation of the patient’s fixed upward gaze. The patient’s home medications included risperidone 0.5mg twice a day, benztropine mesylate 1mg twice a day, and cyanocobalamin 1000mcg daily. His compliance with medication was questionable; however, the patient’s mother reported that the patient took his medications at home. The medical chart noted prior intolerance to haloperidol due to generalized dystonia.

Of note, approximately one month before presentation, the patient was admitted with a diagnosis of dystonia. At that time, he was taking 1mg of risperidone twice daily. After this episode, the patient was prescribed benztropine mesylate, and the dosage of risperidone was tapered down.

His vitals were as follows: temperature 98.4 degrees F, pulse 109 beats per minute, respirations 18 per minute, blood pressure 147/97 mmHg, pulse oximetry 100% on room air. The admission electrocardiogram was unremarkable. Additionally, both computed tomography of the brain without contrast and X-ray of the chest were negative for abnormal findings. The urinary toxicology screen was negative for opiates, methadone, barbiturates, phencyclidine, amphetamines, benzodiazepines, cocaine, and cannabinoids. Urinalysis was also unremarkable. Laboratory results of both complete blood cell count (CBC) and comprehensive metabolic panel (CMP) were without abnormalities.

Given the presentation and medication review, a diagnosis of oculogyric crisis (OGC) was suspected as the most likely differential diagnosis. The patient received 1mg of lorazepam, 25mg of diphenhydramine, and 1mg of benztropine mesylate intravenously. Within minutes of administration of these medications, the patient’s OGC resolved. On repeat examination, the patient was able to move both of his eyes in all directions. At this time, risperidone was discontinued, and a motion for inpatient admission and psychiatric evaluation was initiated.

## Discussion

OGC is a rare neurological condition that was first described in the 1930s in patients with Parkinson’s disease [1]. Extraocular muscles become dystonic, leading to the characteristic deviation of the eyes. Episodes typically last a few minutes, but in some cases, they can persist for many hours [2]. Symptoms may be isolated or may occur in the setting of other forms of dystonia, such as blepharospasm, neck flexion, or tongue protrusion. Although most commonly associated with the use of first-generation antipsychotics, OGCs are known to occur in many other classes of medications, as well as in various neurogenerative disorders and focal brain lesions [3,4]. Risk factors associated with OGC include male sex, younger age, and a family history of neurological disease [2]. OGC remains a clinically relevant topic, as it can be a significant source of distress in patients and can complicate the treatment of psychiatric disorders.

The pathophysiology of OGCs is thought to be due to the disruption of dopaminergic neurotransmission in the nigrostriatal pathway [2]. First, the classes of medications that are most frequently associated with OGCs, such as antiemetics or antipsychotics, often act by disrupting dopaminergic transmission. Additionally, the pathogenesis of neurological disorders that are associated with OGCs, such as Parkinson’s disease or Wilson’s disease, are related to the disruption of dopamine synthesis [4,5]. Finally, in patients who have OGCs in the setting of focal brain lesions, damage to the midbrain or basal ganglia, where the nigrostriatal pathway lies, has been reported [2]. Current research suggests that the extraocular muscle dystonia seen in OGCs is due to increased cholinergic input, as there is insufficient striatal dopaminergic input to suppress cholinergic tone. Unopposed cholinergic stimulation can lead to excessive excitation in medium spiny neurons, causing dystonic symptoms [6]. Indeed, the administration of anticholinergic drugs can lead to the resolution of OGCs.

OGCs are diagnosed clinically based on the presence of characteristic symptoms Table 1 [2]. Once a diagnosis of OGC is suspected, other clinical entities that look similar to OGC must be ruled out. Seizures differ from OGCs in that seizures are typically associated with altered awareness. Ocular dyskinesias last for a few seconds, and patients remain unaware of their condition, whereas OGCs last for minutes to hours and cause significant discomfort or distress. Ocular tics are brief and can be voluntarily suppressed, whereas OGCs cannot be suppressed [5]. Once other causes of eye deviations are ruled out, the cause of the OGC must be investigated. As the majority of OGC cases are drug-induced, a careful review of the patient’s medication list must be performed. Most cases of drug-induced OGCs are due to antipsychotics, and first-generation antipsychotics have a higher incidence of OGCs than second-generation antipsychotics [2]. Antiemetics, anticonvulsants, and antidepressants are also known to cause OGCs [7-9]. If a neurological disorder or focal brain lesion is suspected as the cause of OGC, then appropriate imaging and molecular testing should be performed.

**Table 1:** Diagnostic criteria for oculogyric crisis

Required criteria	Supportive criteria
Dystonic deviation of the eyes	Patient is distressed by the ocular deviations
Preserved consciousness	Preceded by anxiety
Duration lasting minutes to hours	Associated dystonia (e.g. neck flexion)
	Improvement with administration of anticholinergic or dopaminergic medications

Treatment strategies depend on the etiology of the OGC. In drug-induced OGCs, the offending agent should be discontinued. If the medication cannot be discontinued, then the dose should be reduced. OGCs usually resolve within 24-48 hours of cessation or dose reduction of the medication. In acutely severe cases, patients can receive an anticholinergic or an antihistamine intravenously, which leads to alleviation of symptoms in minutes [10]. Patients should then take an oral anticholinergic medication for four to seven days to prevent re-occurrence [10]. If a patient’s symptoms do not improve with these medications, then a benzodiazepine can be given, either orally or intravenously [2]. A few case reports have shown that the administration of L-dopa improves OGCs in patients with parkinsonism, although more research is needed to verify the efficacy of this treatment [11]. Isolated cases have also shown that patients who have OGCs due to focal brain lesions would benefit from anticholinergic or antihistamine therapy [2].

Our patient is unique in that his OGC occurred due to the use of a second-generation antipsychotic. As stated above, first-generation antipsychotics are much more likely to cause OGC than second-generation antipsychotics [2]. A literature search uncovered 22 publications related to OGC in the setting of second-generation antipsychotics. Although much rarer than OGCs that occur from first-generation antipsychotics, the treatment of OGCs due to second-generation antipsychotics remains the same [2]; thus, patients should be monitored closely during treatment for the development of OGC or any other side effects.

Another distinguishing feature of our patient’s presentation is that he had an OGC while on an anticholinergic, which had been prescribed to prevent extrapyramidal symptoms. As the patient was on a low dose of benztropine, it is worth hypothesizing whether the response of OGCs to treatment is dose-dependent. To date, there are no studies investigating whether a dose-dependent relationship exists in the treatment of OGCs. Our patient’s OGC resolved with the administration of additional benztropine, as well as an antihistamine and benzodiazepine. Since all these medications were given at the same time, it is difficult to delineate whether the resolution of this patient’s OGC was due to the higher dose of benztropine or whether it was due to the antihistamine or benzodiazepine. Further clinical studies need to be performed to elucidate whether there exists a dose-dependent response of OGCs to treatment.

## Conclusions

Herein, we have discussed a unique case of a patient with OGC with concomitant second-generation antipsychotic use. This case demonstrates the complexity of antipsychotic medications and the necessity of a thorough review of home medications when investigating for etiology of OGC. It also highlights the established management of this condition and the ease of its reversibility. Ultimately, this case elucidates the intricacies of a possible side effect of second-generation antipsychotics. It demonstrates the need for further research into whether a dose-dependent response exists in order to improve the management of those with a history of OGC in the setting of antipsychotic use.
